# Cyclophilin A Isomerisation of Septin 2 Mediates Abscission during Cytokinesis

**DOI:** 10.3390/ijms241311084

**Published:** 2023-07-04

**Authors:** Rebecca L. Gorry, Kieran Brennan, Paul T. M. Lavin, Tayler Mazurski, Charline Mary, David Matallanas, Jean-François Guichou, Margaret M. Mc Gee

**Affiliations:** 1 School of Biomolecular and Biomedical Science (SBBS), Conway Institute, University College Dublin, D04 V1W8 Dublin, Irelandk.brennan@ucd.ie (K.B.);; 2 Centre de Biologie Structurale, CNRS, INSERM, University Montpellier, 34090 Montpellier, France; 3 Systems Biology Ireland (SBI), School of Medicine, University College Dublin, D04 V1W8 Dublin, Ireland

**Keywords:** cyclophilin, prolyl isomerase, cytokinesis, cell division, cancer

## Abstract

The isomerase activity of Cyclophilin A is important for midbody abscission during cell division, however, to date, midbody substrates remain unknown. In this study, we report that the GTP-binding protein Septin 2 interacts with Cyclophilin A. We highlight a dynamic series of Septin 2 phenotypes at the midbody, previously undescribed in human cells. Furthermore, Cyclophilin A depletion or loss of isomerase activity is sufficient to induce phenotypic Septin 2 defects at the midbody. Structural and molecular analysis reveals that Septin 2 proline 259 is important for interaction with Cyclophilin A. Moreover, an isomerisation-deficient EGFP-Septin 2 proline 259 mutant displays defective midbody localisation and undergoes impaired abscission, which is consistent with data from cells with loss of Cyclophilin A expression or activity. Collectively, these data reveal Septin 2 as a novel interacting partner and isomerase substrate of Cyclophilin A at the midbody that is required for abscission during cytokinesis in cancer cells.

## 1. Introduction

Telophase and cytokinesis mark the final stages of mitosis in which a cell containing duplicated genetic material must elongate and ingress at the cell equator to allow for the final severing of the intercellular bridge and subsequent separation of the two new daughter cells. Following cleavage furrow ingression and constriction of an actinomyosin contractile ring, the two new cells remain tethered by an intercellular bridge approximately 1–3 μm in thickness, which was composed of anti-parallel overlapping bundles of microtubules that contain remnants of the contractile ring [1,2,3]. The central region of the bridge, where the plus-ends of the microtubules overlap, is known as the Flemming body or, more commonly known now, as the midbody [4]. The midbody acts as a platform for the recruitment and assembly of proteins needed for cytokinetic abscission such as CEP55, ESCRT-I and ESCRT-III machinery [5]. Formation of the midbody is essential for abscission, and although over 200 proteins have been shown to localise to the midbody in human cells, knowledge of how most of these proteins are organised, as well as their regulation and function, remains limited [6].

Septins are a family of evolutionarily conserved cytoskeletal GTPases that belong to the same class as the Ras GTPases [7] and are capable of filament formation [8]. Human cells possess 13 septin genes (Sept1–Sept12 and Sept14) [9,10]. One member, Septin 2 (Sept2), is implicated in a wide range of cellular processes, such as cell stiffness and plasma membrane rigidity in yeast [11] and chromosome segregation [12], microtubule regulation and actin dynamics [13], as well as cytokinesis [14] in mammalian cells. During mitosis, Sept2 is recruited to the site of cleavage furrow ingression through its interaction with anillin, where it promotes the formation of septin filaments that associate with the actinomyosin contractile ring during plasma membrane ingression [1,15]. Multiple septins also localise to the midbody, including the palindromic oligomer composed of Sept2, Sept6, Sept7 and Sept9, which forms a double-ring structure that flanks the central midbody and marks the localisation site of ESCRT machinery needed for abscission [16]. However, the regulation of septin ring formation at the midbody remains poorly understood.

Peptidyl-prolyl isomerases (PPIases) are a family of ubiquitous proteins conserved across eukaryotic and prokaryotic species [17] that catalyse the alteration of proline peptide bonds from the cis to trans conformation, and vice versa, thereby acting as a molecular switch in signal transduction cascades [18]. Mammalian cells contain over thirty PPIase genes that are divided into three structurally distinct groups: the FK-506 binding proteins (FKBPs), parvulins and cyclophilins. PPIases have been implicated in a range of diverse functions including protein folding, calcium signalling, transcription [19,20,21,22,23], apoptosis, hypoxia [19,24] and cell division [25,26,27]. The first isomerase to be discovered was Cyclophilin A (CypA), which was identified as the binding partner of the immunosuppressive drug cyclosporin A (CsA) [28]. CypA is a ubiquitous 18 kDa protein composed of eight anti-parallel β sheets forming a right-handed β-barrel with two α-helices that pack against the barrel [29]. It is 165 amino acids in length and contains a conserved cyclophilin-type domain between amino acids 2–163, which form the enzymatic pocket responsible for binding to proline-containing peptides [24].

To date, multiple studies have highlighted the association of CypA over-expression and tumourigenesis in humans, including haematopoietic [26], lung [30], endometrial [31] and prostate cancer [32], and CypA has been proposed as a therapeutic target [24,25]. CypA has been shown to localise to the centrosome in human tumour cells. Furthermore, CypA is displaced from the centrosome and re-locates to the midbody during cytokinesis, where it plays a role in timely abscission [25,26]. Loss of CypA expression led to delayed cytokinesis, supernumerary centrosomes and genome instability and importantly reduced colony formation, demonstrating its importance for the maintenance of tumour growth. Investigation into its mechanism of action revealed that CypA isomerase activity is essential for timely cytokinetic completion [26]. However, isomerase substrates of CypA that play a role in abscission remain to be identified. The identification of CypA substrates is challenging due to the transient nature of peptidyl-prolyl isomerisation, whereby the interconversion of a proline bond from the cis to trans conformation takes place over milliseconds [33,34]. The identification of midbody substrates of CypA is further compounded by the short time during cytokinesis when CypA is localised to the midbody of a dividing cell [25,26]. Despite this, other studies have reported a role for the Pin1 isomerase during cytokinesis through the regulation of Sept9 [35] and CEP55 [27].

In this study, we outline the pattern of Sept2 localisation at the midbody in mammalian cells. We demonstrate that Sept2 is a novel interacting partner of CypA, and that the localisation of Sept2 to the central midbody is dependent on CypA isomerisation. Finally, we reveal Sept2 Pro259 as a novel site of CypA isomerisation required for abscission during cytokinesis.

## 2. Results

### 2.1. Sept2 Is a Novel Interacting Partner of CypA

**Figure 1:**
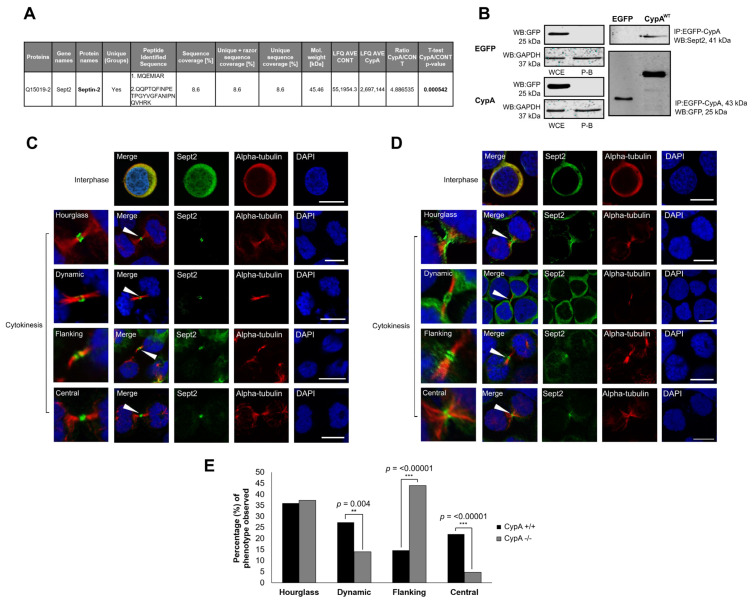
CypA interacts with Sept2, and loss of CypA expression significantly delays Sept2 midbody flanking-to-central transition during cytokinesis. (**A**) Table summarising the results obtained for Sept2 by label-free quantification (LFQ) MaxQuant analysis of the CypA interactome. The coverage of the protein is 8.6%. ‘CONT’ refers to the values obtained for the EGFP immunoprecipitation control, while ‘CypA’ refers to the values obtained for the EGFP-CypA^WT^ immunoprecipitation. Fold change was calculated by dividing the average ‘CONT’ values by the average ‘CypA’ values. *p*-values were derived by *t*-tests using values obtained for three experimental replicates and two technical replicates. K562 cells were transfected with pEGFP and pEGFP-CypA^WT^ (**B**) and lysed 48 h post-transfection, and exogenous EGFP-tagged proteins were immunoprecipitated using GFP-Trap beads and resolved by SDS-PAGE. Resolved proteins were analysed by Western blot using an anti-Sept2 primary antibody. Whole-cell extracts (WCE) and post-binds (P-B) collected after immunoprecipitation were probed with anti-GFP to confirm the presence and absence of exogenous EGFP. Jurkat^CypA+/+^ (**C**) and Jurkat^CypA−/−^ (**D**) cells were enriched in mitosis by treatment with nocodazole (160 nM) for 16 h before being released into complete media until they reached the midbody stage. Cells were collected by cytocentrifugation and immunostained with anti-Sept2 and anti-α-tubulin primary antibodies followed by incubation with AlexaFluor 488 and 594 secondary antibodies, respectively. Cells were counterstained with DAPI and visualised using a 60× oil objective lens (NA 1.4) on an Olympus Fluoview FV1000 confocal microscope. The images are representative of *n* = 150 cells over 3 independent experiments. White arrows indicate the location of the midbody in each image and the location of each region magnified in the left-most panels of (**C**,**D**). Scale bar: 10 µm. (**E**) Bar graph representing the percentage of each Sept2 phenotype observed at the midbody in Jurkat^CypA+/+^ and Jurkat^CypA−/−^ cells following midbody enrichment using nocodazole treatment. The graph represents the percentage of 150 cells analysed per condition. Differences observed between the flanking and central midbody phenotypes were compared, and *p*-values were derived from one-sample, two-tailed Z-tests. Asterisks are indicative of statistical significance (** = *p*-value < 0.01, *** = *p*-value < 0.001).

Loss of CypA expression or isomerase activity is sufficient to induce multinucleation and supernumerary centrosomes as a result of delayed or incomplete cytokinesis [26]. Antibody interference of Sept2 during mitosis has previously been reported to induce mitotic defects of a similar nature [36], suggesting that these two proteins may act in a similar manner or pathway during division. A possible functional relationship between these two proteins was further supported by interaction proteomic screening using EGFP-CypA^WT^ as bait. Upon statistical analysis, it was found that Sept2 is a novel putative interacting partner of CypA (Figure 1A). Considering this, as well as previous reports that CypA and Sept2 localise to the midzone and midbody in mammalian cells [16,26], the ability of CypA to directly interact with Sept2 was investigated by co-immunoprecipitation assays. Results reveal that endogenous Sept2 interacts with exogenous EGFP-CypA^WT^ (Figure 1B), thereby validating Sept2 as a novel interacting partner of CypA.

### 2.2. Localisation of Sept2 at the Midbody in Human Cancer Cells

Septins play crucial roles in the organisation and assembly of the contractile actomyosin ring at the cell equator during mitosis, which promotes initial intercellular bridge formation [37,38]. Within the intercellular bridge, Sept2 forms a filamentous double-ring structure flanking the midbody in Madin–Darby canine kidney (MDCK) epithelial cells in a complex with Sept6, Sept7 and Sept9 [16]. The double-ring structure observed is similar to that reported for septins localised to the site of budding in *Saccharomyces cerevisiae* [39,40]. However, to date, the precise organisation of septins at the site of abscission in mammalian cells remains poorly characterised. In this study, Sept2 localisation at the midbody was examined in Jurkat human T-lymphoma cells that express CypA.

Cells were enriched at the midbody stage of the cell cycle by nocodazole block, followed by a subsequent release to allow mitotic progression. Results reveal four distinct Sept2 phenotypes at the midbody during cytokinesis in Jurkat^CypA+/+^ cells, three of which resemble that observed in *S. cerevisiae* at the site of the budding neck [40]; an ‘hourglass’ phenotype where Sept2 is oriented parallel to the intercellular bridge; a ‘dynamic’ phenotype where Sept2 undergoes disassembly from the hourglass orientation and a ‘flanking’ phenotype where Sept2 re-organises at a 90° angle to the hourglass structure and flanks the central midbody. Finally, the Jurkat^CypA+/+^ cells exhibit a fourth Sept2 phenotype; a ‘central’ phenotype was observed where Sept2 localises at the central midbody (Figure 1C). These results reveal that Sept2 localisation at the midbody in human cells resembles that of septin organisation at the budding neck in *S. cerevisiae* [39,40]. Sept2 localisation at the midbody in Jurkat cells also resembles that of other septin family members in MDCK epithelial cells and HeLa cells, as well as the septin-interacting partner anillin in HeLa cells [1,16,41]. Collectively, these data highlight the similarities between the well-characterised septin cycle in *S. cerevisiae* and the cycle of midbody localisation observed within mammalian cells including human cancer cells. Moreover, this study reveals the transition of Sept2 from a midbody-flanking position to central midbody localisation in human cancer cells; a phenotype that has not been reported previously in yeast or mammalian cells.

### 2.3. Depletion of CypA Disrupts Sept2 Localisation at the Midbody

Data so far reveal an interaction between CypA and Sept2 (Figure 1A,B). Considering the midbody localisation of these two proteins [25,26] (Figure 1C), the possibility that CypA plays a role in Sept2 dynamics at the midbody was investigated. To do that, Sept2 localisation was examined in Jurkat cells depleted of CypA by homozygous knock-out (Jurkat^CypA−/−^) (Figure 1D). Upon examination by confocal microscopy, it was found that Jurkat^CypA−/−^ cells exhibited all four Sept2 midbody phenotypes described in the Jurkat^CypA+/+^ cells (Figure 1C). A similar level of Sept2 with an hourglass orientation was detected in the WT and CypA^−/−^ cells. In contrast, a highly significant increase in midbody-flanking Sept2 (*p*-value < 0.00001) and a corresponding decrease in Sept2 at the central midbody (*p*-value < 0.00001) were detected relative to the Jurkat^CypA+/+^ cells (Figure 1E). A reduction in the transitionary dynamic state was also observed following the loss of CypA expression, which is consistent with a build-up of Sept2 in the midbody flanking state. Collectively, these data reveal that loss of CypA expression in Jurkat cells alters Sept2 dynamics at the midbody and suggest that Sept2 cannot locate to the central midbody and instead builds up in a midbody flanking orientation.

### 2.4. Inhibition of CypA Isomerase Activity Alters Sept2 Dynamics at the Midbody

**Figure 2:**
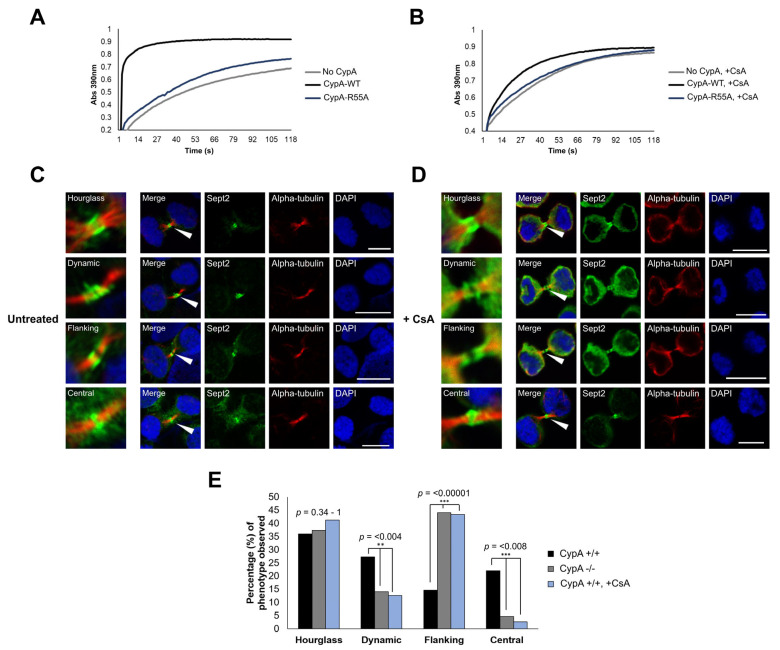
(**A**) Chymotrypsin cleavage of the substrate N-succinyl-Ala-Ala-Pro-Phe-pNitroanilide was quantified in the absence of CypA (‘No CypA’), or presence of His-CypA^WT^ and His-CypA^R55A^, with the addition of CsA (60 nM) to each reaction in (**B**) only. The absorbance of pNitroaniline was measured at 390 nm. The results shown are mean values obtained for each time point across three independent experiments. (**C**,**D**) Jurkat^CypA+/+^ cells were enriched in mitosis following treatment with nocodazole (160 nM) for 16 h before being released into media supplemented with CsA (10 µM) for 60 min prior to collection by cytocentrifugation and immunostaining. All cells were immunostained with anti-Sept2 and anti-α-tubulin primary antibodies, followed by incubation with AlexaFluor 488 and 594 secondary antibodies, respectively. Cells were counterstained with DAPI and visualised using a 60× oil objective lens (NA 1.4) on an Olympus Fluoview FV1000 confocal microscope. The images are representative of *n* = 150 cells over 3 independent experiments. White arrows indicate the location of the midbody in each image and the location of each region magnified in the left-most panels of (**C**,**D**). Scale bar: 10 µm. (**E**) Bar graph representing quantification of Sept2 dynamics at the midbody with hourglass, dynamic, flanking and central phenotypes indicated in Jurkat^CypA+/+^ or Jurkat^CypA−/−^ cells in the absence or presence of CsA (10 μM), as outlined. The graph represents the percentage of 150 cells analysed per condition. Differences observed between the flanking and central midbody phenotypes were compared, and *p*-values were derived from one-sample, two-tailed Z-tests in comparison to untreated Jurkat^CypA+/+^ cells. *p* = <0.00001–1 indicates the range of *p*-values calculated relative to CypA^+/+.^. Asterisks are indicative of statistical significance (** = *p*-value < 0.01, *** = *p*-value < 0.001).

Previously, Bannon et al. demonstrated the importance of CypA isomerase activity in the completion of cytokinesis [26]. Considering the impact of CypA depletion on the Sept2 midbody localisation revealed in this study, the role of CypA catalytic activity in the regulation of Sept2 dynamics at the midbody was investigated using the cyclophilin isomerase inhibitor Cyclosporin A (CsA) [42]. First, the ability of CsA to inhibit the isomerase activity of recombinant His-tagged CypA (His-CypA^WT^) was confirmed using an in vitro α-chymotrypsin-based assay and the synthetic substrate N-succinyl-Ala-Ala-Pro-Phe-pNitroanilide as previously described [43]. His-CypA^WT^ accelerated the substrate cleavage, whereas the His-CypA^R55A^ isomerase inactive mutant did not (Figure 2A). Moreover, the rate of His-CypA^WT^ substrate cleavage was reduced in the presence of CsA to levels similar to that observed with isomerase inactive His-CypA^R55A^ and the negative control (‘No CypA’) (Figure 2B), confirming the isomerase inhibiting activity of the compound. Next, midbody-enriched Jurkat^CypA+/+^ cells were prepared as before (Figure 2C) and treated with CsA (Figure 2D) following release from nocodazole, and cells were prepared for immunofluorescent imaging. Results reveal that treatment of cells with CsA induced a significant increase in Sept2 with a midbody-flanking localisation (*p* < 0.00001) and with a corresponding significant decrease in Sept2 at the central midbody (*p* < 0.00001) when compared to untreated Jurkat^CypA+/+^ cells (Figure 2E), which is consistent with data observed in Jurkat^CypA−/−^ cells (Figure 1E). Taken together, these results suggest that CypA isomerase activity is required for the transition of Sept2 from a midbody flanking to central localisation.

### 2.5. Isomerisation of Pro259 Is Required for Central Midbody Localisation of Sept2

**Figure 3:**
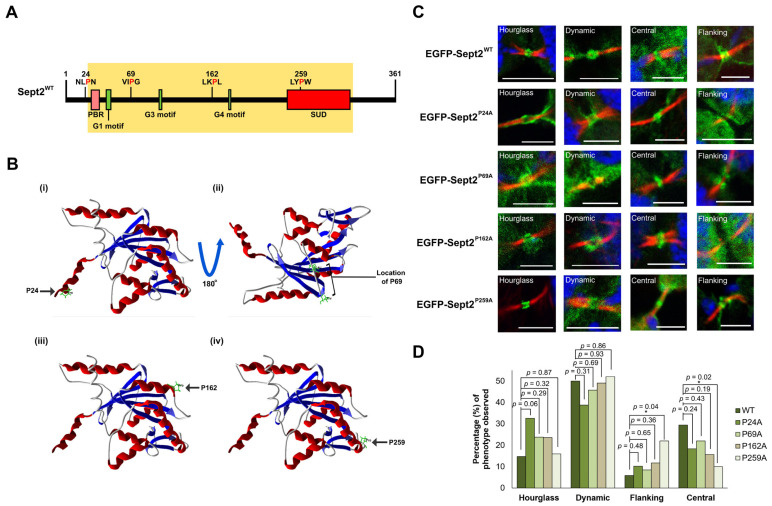
(**A**) Schematic representing the full-length sequence of Sept2, displaying the GTPase domain (yellow box) and the four putative CypA isomerisation sites at Pro24, Pro69, Pro162 and Pro259. PBR: phosphoinosite-binding polybasic region, G1–4: guanine nucleotide-binding motifs, SUD: septin-unique domain [48]. (**B**) Molecular modelling of Sept2 using Molegro Molecular Viewer (http://molexus.io/molegro-molecular-viewer/,) (accessed on 13 November, 2019) with the trimer Sept2-Sept6-Sept7 crystal structure determined by Sirajuddin, M (PDB ID: 2QAG) [49]. Pro24 is part of an exposed α-helix (**i**); Pro69 is located within an external loop between two α-helices (**ii**); Pro162 is located within an α-helix (**iii**) and Pro259 is located within a loop (**iv**). (**C**) K562 cells were transfected with pEGFP-Sept2^WT^, pEGFP-Sept2^P24A^, pEGFP-Sept2^P69A^, pEGFP-Sept2^P162A^ or pEGFP-Sept2^P259A^ and enriched in mitosis by nocodazole treatment (160 nM) for 16 h followed by release into complete media for 60 min prior to cytocentrifugation and immunostaining. All cells were immunostained with anti-α-tubulin primary antibody, followed by incubation with AlexaFluor 594 secondary antibody. All cells were counter-stained with DAPI and visualised using a 60× oil objective lens (NA 1.4) on an Olympus Fluoview FV1000 confocal microscope. Images are representative of the percentage of 50 cells per condition over 3 independent experiments. Scale bar: 5 μm. (**D**) Bar chart highlighting the percentage (%) of each midbody phenotype observed within the five cell populations. Differences observed between the flanking and central midbody phenotypes were compared, and *p*-values were derived from one-sample, two-tailed Z-tests in comparison to EGFP-Sept2^WT^ cells. Asterisks are indicative of statistical significance (* = *p*-value < 0.05).

In this study, Sept2 has been confirmed as a CypA-interacting protein, and loss of CypA expression, or enzymatic activity, alters Sept2 dynamics at the midbody, suggesting that CypA-mediated isomerisation regulates Sept2 localisation, and thereby function, at the midbody. CypA binds to substrates such as the HIV-1 capsid (CA) protein and the cell surface receptor cluster of differentiation protein CD147 through a short motif consisting of [H-X-P-H], where H denotes any hydrophobic amino acid and X indicates any amino acid [44,45,46]. Sept2 contains 14 proline residues that were investigated as potential CypA isomerisation sites using a number of criteria including the identity of surrounding amino acids, accessibility and evolutionary conservation (Supporting Information, Table S1). Five conserved proline residues were located within the proposed CypA consensus motif: Pro69, Pro155, Pro162, Pro179 and Pro259 (Figure 3A). Structural analysis revealed surface localisation of Pro69, Pro162 and Pro259, suggesting that they are amenable to protein–protein interaction (Figure 3B). Pro24 is evolutionarily conserved and is located on the protein surface; however, it does not lie within the proposed CypA binding motif. Therefore, it is considered less likely to be an isomerisation substrate (Supporting Information, Table S1, Figure 3B). Nevertheless, the four candidate sites, Pro24, Pro69, Pro162 and Pro259, were prioritised for investigation as potential CypA isomerisation residues within Sept2.

Mutation of individual proline residues to alanine within CypA substrates is an effective approach to investigate the functional significance of proline isomerisation [45,47]. pEGFP-Sept2 isomerase-defective mutants (Pro24Ala, Pro69Ala, Pro162Ala and Pro259Ala) were generated and expressed in human leukemic K562 cells. Midbody localisation of each Sept2 mutant, as well as EGFP-Sept2^WT^, was examined by confocal microscopy (Figure 3C). EGFP-Sept2^WT^ and each Sept2 proline mutant were capable of localising to the intercellular bridge. Importantly, EGFP-Sept2^WT^ exhibited a pattern of localisation similar to endogenous Sept2 (Figure 1C) and underwent hourglass, dynamic, flanking and central localisation. EGFP-Sept2^P24A^, EGFP-Sept2^P69A^, EGFP-Sept2^P162A^ and EGFP-Sept2^P259A^ exhibited similar phenotypic distribution in regard to the hourglass and dynamic Sept2 localisation, while EGFP-Sept2^P24A^, EGFP-Sept2^P69A^ and EGFP-Sept2^P162A^ also showed similar ratios of central and flanking localisation when compared to EGFP-Sept2^WT^ (Figure 3D). In contrast, EGFP-Sept2^P259A^ exhibited a significant increase in midbody-flanking localisation (*p*-value: 0.046) with a corresponding decrease in central localisation (*p*-value: 0.022) when compared to EGFP-Sept2^WT^ (Figure 3D). Moreover, the pattern of localisation of EGFP-Sept2^P259A^ is similar to endogenous Sept2 localisation in cells lacking CypA (Jurkat^CypA−/−^ cells) (Figure 1E) and in wild-type cells where CypA isomerase activity has been inhibited by treatment with CsA (Figure 2E). Collectively, this data suggests that Sept2 Pro259 is a site of CypA isomerisation.

### 2.6. Structural and Molecular Analysis of CypA-Sept2^P259A^ Interaction

**Figure 4:**
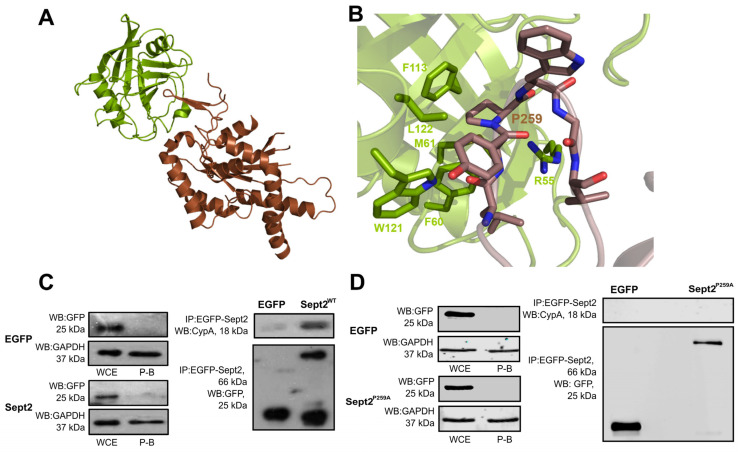
Sept2-Pro259 is critical for the interaction between Sept2 and CypA. (**A**) Cartoon representation of the modelling performed for the interaction between CypA (green) and Sept2 (brown). (**B**) Zoomed in view of the active site of CypA (green) with regards to Pro259 of Sep2 (brown). K562 cells were transfected with pEGFP, pEGFP-Sept2^WT^ (**C**) or pEGFP-Sept2^P259A^ (**D**); lysed 48 h post-transfection and exogenous EGFP-tagged proteins were immunoprecipitated using GFP-Trap^®^ beads and resolved by SDS-PAGE. Resolved proteins were analysed by Western blot using anti-CypA (**C**,**D**) primary antibodies. Whole-cell extracts (WCE) and post-binds (P-B) collected after immunoprecipitation in (**C**,**D**) were probed with anti-GFP to confirm the presence and absence of exogenous EGFP.

CypA binds directly to proline residues within substrates to catalyse their isomerisation from the cis-to-trans conformation and vice versa, and mutation of these residues is sufficient to abrogate the interaction. For example, Yurchenko et al. demonstrated that mutation of Pro211 to alanine prevented the interaction between CypA and CD147 [45]. Data so far reveal the importance of CypA isomerase activity in Sept2 midbody dynamics (Figure 2) and highlight Sept2 Pro259 as a candidate site of CypA isomerisation required for flanking-to-central midbody transition (Figure 3). To support this further, the ability of CypA to interact specifically with Sept2 through P259 was investigated by structural and molecular analysis. Molecular modelling of the interaction between the two proteins, whereby both the CypA active site Arg55 and Sept2 Pro259 were present (Figure 4A), revealed, with confidence, an interaction between CypA and Sept2 via Pro259. Residues Leu257 to Val262 of the Sept2 binding loop are located within the binding groove of CypA and make extensive hydrophobic interactions at the interface, while the key catalytic arginine within CypA, Arg55, is in interaction with Sept2 Pro259 (Figure 4B). The model presented in Figure 4B highlights the orientation and close proximity (3.5 Å) of the CypA guanidine side chain to Sept2 Pro259, which likely facilitates stabilisation of the proline nitrogen during catalysis [50]. The carbonyl group at Sept2 Leu257 also contributes to the interaction interface through the NƐ1 of the CypA ring containing Trp121.

The importance of Pro259 for the interaction was further demonstrated by co-immunoprecipitation, whereby mutation of Pro259 to alanine completely abolished the interaction between EGFP-Sept2 and CypA (Figure 4C,D). Taken together with the imaging data obtained as part of this study, these results highlight the importance of Pro259 in the interaction between CypA and Sept2 and in the regulation of Sept2 localisation at the central midbody.

### 2.7. Isomerisation of Sept2 Pro259 Is Required for the Completion of Cell Division

**Figure 5:**
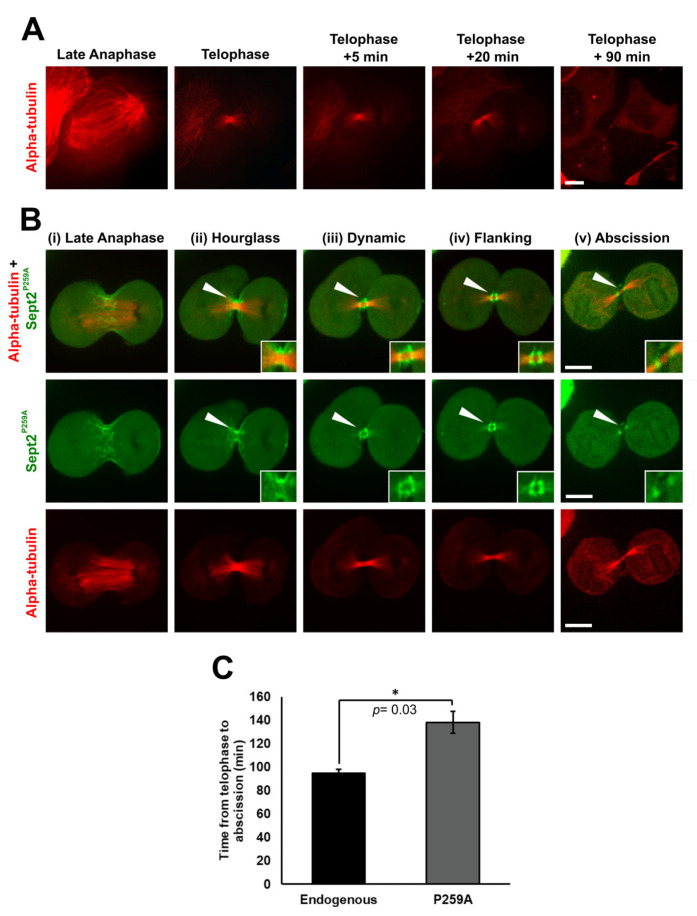
Expression of EGFP-Sept2^P259A^ in HeLa cells impairs abscission. (**A**,**B**) HeLa cells were transfected with pmCherry-α-tubulin alone (**A**) or with pmCherry-α-tubulin and pEGFP-Sept2^P259A^ (**B**) before mitotic enrichment by nocodazole treatment (160 nM) for 16 h. Cells were released from nocodazole and imaged immediately using a Nikon Eclipse Ti-E microscope equipped with a temperature controlled, humidified chamber (37 °C, 5% CO_2_) and an 100× oil-immersion objective lens (1.4) coupled with intermediate magnification (1.5×). Z-stacks (0.3 μm slices, 20 μm in total) were acquired from metaphase/late anaphase onwards, every 60 s to 80 s for up to 150 min post-telophase. The white arrow indicates the location of the ingressed midzone and subsequent midbody within the intercellular bridge. (i) to (v) indicate the stages of Sept2 dynamics depicted during cytokinetic progression. Scale bar: 15 μm. (**C**) Quantification of time from telophase to abscission of cells expressing Sept2^P259A^ relative to cells expressing endogenous Sept2 (date represents mean time from 3 independent live cell recordings for each condition). Differences observed in time to abscission were compared, and *p*-values were derived from a two-tailed, two-sample *t*-test assuming unequal variance. Asterisks are indicative of statistical significance (* = *p*-value < 0.05).

It is previously reported that loss of CypA expression significantly delays Jurkat cell division [26]. In this study, it has been shown that Sept2 is an interacting partner of CypA (Figure 1A,B and Figure 4C), and that CypA isomerisation of Pro259 is essential for central midbody localisation of Sept2 (Figure 3C,D). Thus, it was postulated that a delay or inhibition in the transition of Sept2 to the central midbody may manifest in delayed or stalled cytokinesis. Therefore, the effect of over-expressing EGFP-Sept2P259A on time to cytokinetic completion of HeLa cells was examined by live cell imaging. It was found that cells expressing pEGFP-Sept2^WT^ exhibited mitotic defects such as multipolarity and arrest at the metaphase/anaphase transition (Supporting Information, Figure S1 and Video S3), revealing that expression of exogenous EGFP-Sept2^WT^ together with endogenous Sept2 in the HeLa cells induces mitotic defects and CIN, which is possibly due to a disruption in the balance of optimal Sept2 levels within the cell that is required for normal cytokinesis. Consistent with that, over-expression of Sept2 has been reported in multiple cancer types and shown to facilitate tumourigenesis [51,52,53]. Therefore, HeLa cells expressing mCherry-α-tubulin alone were included as a control for the measurement of time to abscission in cells expressing wild-type endogenous Sept2 and cells expressing Sept2^P259A^. HeLa cells expressing mCherry-α-tubulin alone undergo abscission 90 min post-telophase (Figure 5A, Supporting Information and Video S1), whereas cells expressing EGFP-Sept2^P259A^ and mCherry-α-tubulin display defects in localisation similar to that observed earlier in this report for both the Jurkat^CypA−/−^ and K562 cells (Figure 5B, Supporting Information and Video S2). Telophase was identified as the point at which cleave furrow ingression had occurred, and the α-tubulin present at the midzone had compacted to form a distinct intercellular bridge between the new daughter cells. Specifically, during late anaphase, EGFP-Sept2^P259A^ displayed a filamentous structure at the plasma membrane at the equator of the cell (Figure 5B(i)). At the onset of telophase, EGFP-Sept2^P259A^ remained associated with the plasma membrane in an hourglass structure parallel to the intercellular bridge (Figure 5B(ii)). During telophase progression, EGFP-Sept2^P259A^ displays an intermediate ‘dynamic’ phenotype where it disassociates from the plasma membrane and reassembles as a double-ring midbody-flanking structure (Figure 5B(iii)). Importantly, EGFP-Sept2^P259A^ persists in a midbody-flanking position (Figure 5B(iv)) and does not transition to central midbody localisation. Moreover, cells expressing EGFP-Sept2P259A remained connected by the intercellular bridge 90 min post-telophase (Figure 5B(v)) when control cells expressing mCherry-α-tubulin alone completed abscission. Live imaging reveals that the cells are rounded and tethered by the intercellular bridge up to 150 min post-telophase (Supporting Information, Video S2 and Figure 5C). Overall, these data confirm the importance of Pro259 for Sept2 dynamics at the midbody, including central localisation and the timely completion of cell division.

## 3. Discussion

Cytokinesis is a highly regulated process that is tightly co-ordinated with chromosome segregation during the earlier stages of mitosis. Cytokinetic failure can result in an increase in CIN and the generation of tetraploid and aneuploid cells, thereby promoting tumourigenesis [54]. Furthermore, the loss or over-expression of key proteins involved in cytokinesis such as LATS1, PRC1 or MKLP2 as well as mutations in the mitotic checkpoint complex (MCC) prior to the metaphase/anaphase transition can lead to cytokinetic failure [55,56,57,58,59,60]. CypA is over-expressed in a range of human tumour cells and is associated with enhanced cell proliferation [24,61]. CypA localises to the midbody during cancer cell division and its isomerase activity is required for the timely completion of cytokinesis [26]. Loss of CypA expression in a variety of human cancer cells delayed cytokinesis and reduced colony formation, demonstrating its importance in tumour growth [26,62]. Despite its important function during cell division, the mechanism of action of CypA, or the identity of isomerase substrates at the midbody, remains unknown.

Septins have long been associated with the process of cytokinesis [12,63]; for example, the absence of Sept2 or Sept11 has been shown to induce abnormal cleavage furrow constriction [41]. Sept2, Sept6, Sept7, Sept9 and Sept11 have been shown to localise to the intercellular bridge and in a midbody-flanking position in HeLa and MDCK cells [16,41], suggesting a role for septins in the final abscission step during cytokinesis. In support of this, the depletion of Sept9 led to a delay in the abscission of HeLa cells [41]. Furthermore, the re-localisation of the ESCRT-III component CHMP4B from the midbody to the site of abscission is dependent on anillin–septin interactions [1]. However, despite evidence supporting the important roles of septins during cytokinesis in mammalian cells, their precise functions and mechanisms of action remain poorly understood.

Through mass spectrometry and co-immunoprecipitation, we show that Sept2 is a novel interacting partner of CypA. Given the functions of CypA and septins during cell division, a possible interaction between these proteins at the midbody during cytokinesis was further investigated. Septin dynamics at the site of abscission within the budding neck in *S. cerevisiae* are well characterised in contrast to mammalian cells [39,40]. In *S. cerevisiae*, septin octamer and single filaments arrange in a radial hourglass structure at the site of abscission. During budding, septin structures are disassembled from the hourglass structure and subsequently re-assembled at a 90° angle where they form a double-ring structure that flanks the site of abscission [40]. In mammalian cells, septins have been shown to localise to the spindle midzone and assist in the formation of the actinomyosin contractile ring [16,41]; however, midbody dynamics are not well characterised. In this study, we reveal a sequence of dynamic localisation patterns of Sept2 at the midbody in human cancer cells, which is similar to that described at the site of budding in yeast [40].

Following initial ingression, Sept2 remains associated with the plasma membrane parallel to the intercellular bridge in an hourglass-like phenotype. During intercellular bridge elongation, Sept2 dissociates from the plasma membrane and reassembles as a double-ring structure that flanks the midbody. An intermediate dynamic phenotype was observed during the disassembly and re-assembly of Sept2 filaments within the bridge, which is consistent with that reported in yeast. Finally, it was revealed that Sept2 transitions from the double-ring midbody-flanking position to a central midbody localisation. The hourglass and midbody-flanking phenotype detected in this study are consistent with a recent report in canine epithelial cells [16]; however, this is the first report of septin localisation at the central midbody, which has not been reported previously for septins in yeast or mammals and thus provides new insight into their mechanism of action during cell division. Considering that Sept6, Sept7 and Sept9 are located on the intercellular bridge, it is possible that they also localise to the central midbody; however, that remains to be determined. Furthermore, the central midbody localisation demonstrated in this study may be specific to mammalian cells or to the tumour cells used.

In addition to the central midbody localisation of Sept2 outlined in this study, we demonstrate a dependence on the expression of the prolyl isomerase CypA for the flanking-to-central midbody transition of Sept2. Moreover, Sept2 localisation to the central midbody was blocked by the isomerase inhibitor cyclosporin A, suggesting that central midbody localisation of Sept2 is dependent on proline isomerisation. Further investigation revealed a critical role for Sept2 Pro259 in not only mediating the interaction between Sept2 and CypA, but also in the flanking-to-central transition and ultimately the timely completion of cytokinesis.

Importantly, these data support a model whereby isomerisation of Sept2 at Pro259 induces a conformational change that acts as a molecular switch to mediate the transition of Sept2 from midbody flanking-to-central localisation that is required for abscission during cytokinesis and the generation of two new daughter cells. Although the precise function of central Sept2 remains unknown, it is possible that it is required for the recruitment of factors involved in abscission such as components of the ESCRT-III machinery. In support of this, Karasmanis et al. demonstrated that the localisation of a Sept2–Sept6–Sept7–Sept9 double-ring structure at the midbody demarcates the site of localisation of ESCRT-III machinery needed for membrane fusion and abscission in canine epithelial cells [16]. This raises the question as to whether or not the central transition event of Sept2 described herein is unique to mammalian cells or cancer cells. Furthermore, whether Sept2 alone dissociates from the double-ring Sept2–Sept6–Sept7–Sept9 complex flanking the midbody to localise centrally as a result of its isomerisation by midbody-central CypA, or whether the entire structure localises to the central midbody remains unknown. Karasmanis et al. also demonstrated that Sept9 expression within this flanking ring decreases in MDCK cells as the ESCRT machinery assembles, which could support the idea of the entire structure re-localising to the centre; however, subsequent central localisation of Sept9 was not reported [16]. Thus, it is also possible that isomerisation facilitates protein–protein interactions required for septin filament formation.

Furthermore, while numerous studies demonstrate that loss of septins promotes defective cytokinesis and malignant transformation [51,64,65], results from this study highlight the impact of Sept2-over-expression in HeLa cells, through increased multipolarity and arrest at the metaphase/anaphase transition, suggesting that a precise balance in expression is required for maintenance of genome stability [66].

In conclusion, this study describes a mitosis-specific signalling pathway involving CypA and Sept2, whereby Sept2 is a novel interacting partner and isomerase substrate of CypA at the midbody that is required for cytokinesis. This work adds Sept2 to the list of CypA isomerisation substrates identified so far and is the first midbody substrate to be reported. Furthermore, this study provides new insight into the mechanisms of abscission during cytokinesis, which could be exploited in the development of new targeted approaches to block cancer cell proliferation.

## 4. Materials and Methods

### 4.1. Cell Culture, Synchronisation and Transfection

Jurkat, K562 and HeLa cells were obtained from the European Collection of Cell Cultures (Porton Down, Wiltshire, UK). The Jurkat and K562 lines were grown in RPMI-1640 GlutaMAX medium containing 100µg/mL penicillin/streptomycin and 10% (*v*/*v*) foetal bovine serum (FBS), while the HeLa cells were grown in Minimum Essential Medium (MEM) with the same additives. Midbody enrichment was achieved by treatment of cells with 160 nM nocodazole (Sigma-Aldrich, St. Louis, MO, USA) for 16 h followed by PBS washes before releasing into complete media. Jurkat^CypA−/−^ cells were obtained through the AIDS Research and Reference Reagent Program and were created as described previously by [26].

Plasmid transfections in K562 cells were performed using the Amaxa biosystems Cell Line Nucelofector^®^ Kit V as per the manufacturer’s guidelines, while HeLa cells were transfected with DharmaFECT™ kb transfection reagent (Dharmacon (Lafayette, CO, USA) according to the manufacturer’s instructions for a 96-well set-up.

### 4.2. Proteomics Sample Preparation, Analysis and Identification of Sept2 as a CypA Interactor

K562 cells were transiently transfected with pEGFP or pEGFP-CypA^WT^, lysed 48 h later and subjected to immunoprecipitation as described below (see ‘co-immunoprecipitation’) using GFP-Trap© beads (ChromoTek, GmbH, Germany). Immunoprecipitated proteins were digested as described previously by Turriziani et al., 2014 [67]; in brief, proteins were first incubated in 60 µL Elution Buffer 1 (2 M urea, 50 mM Tris-HCL, pH 7.5, 5 µg/mL trypsin) at 27 °C on a shaker for 30 min. Samples were then subjected to centrifugation at 13,000 RPM for 30 s, and the resulting supernatant was collected. An amount of 25 µL Elution Buffer 2 (2 M urea, 50 mM Tris-HCL, pH 7.5, 1 mM dithiothreitol) was then added to each sample followed by centrifugation at 13,000 RPM for 30 s each, and the resulting supernatant was collected. This step was repeated once more, and both supernatants were combined and incubated at room temperature overnight. Samples were alkylated by the addition of 20 µL iodoacetamide (5 mg/mL) and incubation in the dark for 30 min at room temperature. The reaction was stopped by the addition of 1 µL 100% (*v*/*v*) trifluoroacetic acid to each sample, and 100 µL of each sample was immediately loaded onto equilibrated handmade C18 StageTips containing Octadecyl C18 disks (Supelco, Sigma Aldrich, MO, USA). Equilibration of the tips was achieved using 50 µL 50% (*v*/*v*) acetonitrile and 0.1% (*v*/*v*) TFA, followed by centrifugation and the addition of 50 µL 0.1% (*v*/*v*) TFA. TFA was discarded, and the samples were desalted by 2 × 50 µL 0.1% (*v*/*v*) TFA and eluted with 2 × 25 µL 50% (*v*/*v*) acetonitrile and 0.1% (*v*/*v*) TFA. The resulting eluates were pooled and concentrated using a CentriVap Concentrator (Labconco, MO, USA) to 5 µL, before dilution to a final volume of 15 µL using 0.1% (*v*/*v*) TFA. Samples were centrifuged for 10 min at 13,000 RPM and subjected to Liquid Chromatography-Tandem Mass Spectrometry (LC-MS) using a Nanoflow Ultimate 3000 LC and Q-Exactive mass spectrometer (Thermo Fisher Scientific, Waltham, MA, USA). Three experimental replicates were injected twice, and data were acquired in an automatic data-dependent switching mode, selecting the 12 most intense ions prior to MS/MS analysis.

Raw files and label-free quantification (LFQ) was performed using MaxQuant (Ver. 1.3.0.5). Default settings were used unless stated otherwise: the initial search was set at 20 ppm (main search performed at 6 ppm mass accuracy and 20 mass deviation for fragment ions), data were searched against a human database (Uniprot: HUMAN), a false discovery rate (FDR) of 0.01 was set for both peptides and proteins, a minimum peptide length of 6 was applied and LFQ with a minimum ratio count was set to 1. Specific proteins were identified based on their fold change and *p*-values derived from *t*-test calculations; proteins with a fold change of >2 and *p*-values of <0.01 were considered specific. Fold changes were calculated by dividing the EGFP-CypA^WT^ immunoprecipitation LFQ intensities by the EGFP control LFQ intensities, while *t*-tests were performed using the average LFQ values obtained between the EGFP and EGFP-CypA^WT^ immunoprecipitations.

### 4.3. Co-Immunoprecipitation

Immunoprecipitation of exogenously expressed EGFP-tagged proteins was performed using GFP-Trap© beads according to the manufacturer’s protocol. Mammalian cells were transiently transfected with the relevant EGFP-tagged construct as described and incubated at 37 °C in 5% CO_2_ for a period of 24–72 h. Cells were collected by centrifugation, washed once in PBS and lysed in 200 µL co-IP lysis buffer (10 mM Tris/HCl pH 7.5, 150 mM NaCl, 0.5 mM EDTA, 0.5% (*v*/*v*) NP-40); they were supplemented with 1 mM PMSF, 1 µg/mL aprotinin, 1 µg/mL leupeptin and 1 µg/mL pepstatin A by centrifugation at 30,000× *g* for 30 min at 4 °C. Protein concentration was determined by Bradford assay, and 1.5 mg of protein was diluted with dilution/wash buffer (10 mM Tris/HCl pH 7.5, 150 mM NaCl, 0.5 mM EDTA) to a final volume of 500 µL. GFP-Trap© beads were equilibrated, with a 25 µL bead slurry being used for each pull-down. Equilibrated beads were added to the diluted protein lysates and tumbled end-over-end for a minimum of 1 h at 4 °C. Samples were centrifuged and the resulting post-bind (PB) was transferred to a clean Eppendorf and stored at −20 °C for Western blot analysis. The beads were washed and resuspended in 50 µL 2× sample buffer (8% (*w*/*v*) SDS, 250 mM Tris/HCl, pH 6.8, 40% (*v*/*v*) glycerol, 0.5% (*w*/*v*) Bromophenol blue, 4% (*w*/*v*) β-mercaptoethanol). The samples were boiled at 95 °C for 10 min to dissociate immunoprecipitated complexes from the GFP-Trap© beads. SDS-PAGE and Western blot were used as described below.

### 4.4. Immunoblotting

Cells were resuspended in RIPA buffer (150 mM NaCl, 50 mM Tris/HCl pH 7.4, 0.25% (*w*/*v*) sodium deoxycholate, 1% NP40/IGEPAL), supplemented with 1 mM PMSF, 1 µg/mL aprotinin, 1 µg/mL leupeptin and 1 µg/mL pepstatin A and lysed by centrifugation at top speed in a bench-top centrifuge for 30 min at 4 °C. Protein concentration was determined by Bradford assay; an equal amount of protein (50 µg) was separated by SDS-PAGE (15%), and proteins were transferred onto either polyvinylidene difluoride (PVDF) membranes for traditional ECL development, or onto Immobilon^®^-FL PVDF for LI-COR^®^ development using the Mini-Protean II blotting system (Bio-Rad, Hercules, CA, USA). Membranes were blocked for 1 h at room temperature in 5% (*w*/*v*) bovine serum albumin (BSA) in TBS-T (TBS containing 0.1% (*v*/*v*) Tween 20). Membranes were incubated with the appropriate antibody at 4 °C overnight. After incubation, membranes were washed in TBS-T and incubated with goat anti-rabbit or anti-mouse horseradish peroxidase-coupled secondary antibody diluted 1:2000 in TBS-T containing 5% (*w*/*v*) BSA for 1 h at room temperature for ECL development (Pierce ECL, Thermo Fisher Scientific, MA, USA). For LI-COR^®^ development, membranes were washed in TBS-T and incubated with DyLightTM Fluor 688 (mouse) or 800 (rabbit) conjugate secondary antibodies. Regardless of development, membranes were then washed for 35 min in TBS-T and TBS before using an ECL detection kit according to the manufacturer’s instructions or using the LI-COR^®^ Biosciences Odyssey^®^ Infrared Imaging System.

For a complete list of the antibodies used throughout this study, please refer to Supplementary Information, Table S2.

### 4.5. Immunofluorescence

An aliquot of suspension cells (100 µL) was cytocentrifuged onto glass slides at 700 g for 2 min using a Shandon Cytospin 2 (Thermo Fisher). Cells were fixed with 3% (*w*/*v*) paraformaldehyde in PBS for 30 min at room temperature and washed with PBS. Cells were then permeabilised using 0.1% (*v*/*v*) Triton X-100 in PBS for 3 min at room temperature. After washing with PBS, the cells were blocked in 2% (*w*/*v*) BSA (PBS) for 30 min at room temperature, washed and incubated with the appropriate primary antibody (1:200) for 1 h at 4 °C. After washing in PBS, the cells were incubated with either anti-rabbit or anti-mouse Alexa Fluor 594 or 488 antibodies (1:200 dilution) (Invitrogen, MA, USA), respectively, in 2% (*w*/*v*) BSA (PBS) for 1 h at room temperature in the dark. Nuclei were stained with DAPI (1 µg/mL) for 20 s. Coverslips were fixed to the slides using Dako Cytomation fluorescent mounting medium (Dako Inc. MO, USA). Stained cells were visualised using an Olympus Fluoview FV1000 confocal microscope with a 60× oil objective lens (numerical aperture (NA) 1.4), and images were acquired by FV10-ASW 3.0 software. Pixel dwell time in all experiments was kept at 8 μs/pixel, with Kalman filtering (line, 5 scans) included to decrease background signal or noise. Each channel was acquired sequentially to avoid signal bleed-through; with the 488 nm laser fired first (EGFP), followed by 561/594 nm (red fluorescent co-stain) and finishing with the 340 nm laser (DAPI nuclear signal). Further image analysis was performed using Fiji.

When comparing the four Sept2 phenotypes observed, namely, Hourglass, Dynamic, Flanking and Central, in Figure 1, and Figure 2, 50 cells were imaged blindly per experiment; thus, a total of 150 cells were imaged for each condition (Jurkat^CypA+/+^, Jurkat^CypA−/−^, Jurkat^CypA+/+^, ^+CsA^) over 3 independent experimental set-ups. Any cells that exhibited a distinct, fully-formed intercellular bridge as demonstrated by α-tubulin staining were imaged across 6–8 fields of view on each individual slide, and the percentages of each Sept2 phenotype observed for Jurkat^CypA−/−^ and Jurkat^CypA+/+^, ^+CsA^ cells were statistically compared to that observed for Jurkat^CypA+/+^ cells. Similarly, in Figure 3, a total of 50 cells were imaged for each condition (EGFP-Sept2^P24A^-, EGFP-Sept2^P69A^-, EGFP-Sept2^P162A^-, EGFP-Sept2^P259A^-transfected cells) across 3 independent experimental set-ups, and the percentages of each Sept2 phenotype observed were statistically compared to cells transfected withEGFP-Sept2^WT^.

For a complete list of the antibodies used throughout this study, please refer to Supplementary Information, Table S2.

### 4.6. Data and Image Analysis

Data obtained from this study are presented as mean + standard error of the mean (SEM). Statistical analysis was carried out using Microsoft Excel software (Microsoft Office 16), unless otherwise stated, to perform two-tailed, two-sample *t*-tests assuming unequal variance for organelle localisation comparison, or one-sample, two-tailed Z-tests to determine relevant *p*-values.

### 4.7. Isomerase Assay

Peptidyl-prolyl cis/trans isomerase activity was determined using a protease-coupled assay in a multi-mode CLARIOStar microplate reader (BMG Labtech) equipped with onboard injectors. The synthetic substrate peptide (N-succinyl-Ala-Ala-Pro-Phe-pNitroanilide) was dissolved in lithium chloride (500 mM in tetrafluoroethylene) and used at a final concentration of 80 μM. Prior to injection, the tubing was primed by expelling any air/liquid present followed by washing with H_2_O before finally expelling again and placing the tubing in the substrate peptide solution ready for well injection. Each reaction well was set up in triplicate for each recombinant protein/condition being tested. Wells consisted of 50 μL isomerase buffer (100 mM NaCl, 40 mM HEPES, pH 7.9), 2 μL α-chymotrypsin (1 mM in 2 mM calcium chloride and 1 mM HCl, final concentration of 10 μM), 100 nM of the recombinant protein being assessed, +/− 60 nM CsA and H_2_O to a total volume of 190 μL, allowing for a final 10 μL of the substrate peptide as prepared above. Each reaction well was mixed gently by pipetting prior to substrate injection. The substrate was dispensed directly into each well using one onboard injector to a final reaction volume of 200 μL. After substrate addition, absorbance was read immediately at 390 nm, and readings were taken every 1 s for 120 s. When included, ‘No CypA’ controls contained extra H_2_O in place of recombinant protein.

### 4.8. Plasmids and Site-Directed Mutagenesis (SDM)

For a summary of all plasmids used, refer to Supplementary Information, Table S2. The pEGFP-CypA^WT^ plasmid was created as described by Bannon et al. (2012) [26], while pEGFP-C1 (EGFP) was purchased from BD Biosciences Clontech (Palo Alto, CA, USA). pEGFP-Sept2^WT^ was created by Gibson Assembly (New England Biolabs (Hitchin, UK)) using pET28a (+)-Sept2 as template DNA rather than cDNA. pET28a (+)-Sept2 was kindly donated by Dr. Julio Cesar Pissuti Damalio (Centro de Biotecnologia Molecular Estrutural, Instituto de Física de São Carlos, Universidade de São Paulo, São Paulo, Brazil). pEGFP-Sept2^P24A^, pEGFP-Sept2^P69A^, pEGFP-Sept2^P162A^ and pEGFP-Sept2^P259A^ were created by site-directed mutagenesis (SDM) using Phusion^®^ High-Fidelity DNA Polymerase (M0530, New England Biolabs) using the optimal guidelines provided by the manufacturer. In brief, each PCR reaction contained 5× Phusion^®^ HF Buffer, 10 mM dNTPs, 0.5 µM forward primer, 0.5 µM reverse primer, 3% (*v*/*v*) DMSO, 1.5 mM MgCl_2_, 1U Phusion^®^ DNA Polymerase and 2.5–20 ng plasmid DNA (pEGFP-Sept2^WT^) made up to a final volume of 50µL. Thermocycling conditions were as follows: initial denaturation at 98 °C for 30 s, 16–20 cycles of 98 °C for 5–10 s, 45–72 °C* for 10–30 s and 72 °C for 30 s/kb plasmid, followed by a final extension step at 72 °C for 10 min before holding at 4 °C. The annealing temperature at step * was determined on the basis of each primer set; Tm was either calculated using the New England BioLabs^®^ Tm calculator (found at: https://tmcalculator.neb.com/#!/main) (accessed regularly from May 2017 until November 2019) or chosen using the guidelines listed in the trouble-shooting section of the Phusion^®^ protocol found online at https://international.neb.com/Protocols/0001/01/01/pcr-protocol-m0530. For primer details, see Supplementary Information, Table S3.

The resulting PCR products were digested with DpnI (New England BioLabs^®^) at 37 °C for 1 h, followed by analysis through gel electrophoresis. Successful SDM reactions were ligated using T4 DNA Ligase (Promega, WI, USA) for 5 min at room temperature before chemical transformation into XL1 *Escherichia coli* for subsequent miniprep preparation of the plasmid (QIAprep Spin Miniprep Kit, QIAGEN) and confirmation of mutagenesis by sequencing. All sequencing was performed by Eurofins Genomics.

### 4.9. Modelling of the Interaction between CypA and Sept2

The Protein Data Bank (PDB) files 1CWA (accession number, CypA) and 2QNR (accession number, Sept2) were selected as support to generate a model of protein–protein interaction. Modelling simulation was performed using the server ZDOCK [68] (http://zdock.umassmed.edu/) with only one constraint, Arg55 (CypA) and Pro259 (Sept2) should be present within the interaction (as results, see Figure 4B); from this, 10 models were generated. A visual inspection of the 10 models was performed, and 6/10 were found to show the same orientation for both partners (see Figure 4A). The quality of the model was controlled using QMEAN (https://swissmodel.expasy.org/qmean/) [69] with a score of 0.95, and a manual inspection was performed to ensure the quality of the interaction predicted. The pictures for proteins were generated using Pymol.

### 4.10. Time-Lapse Spinning Disk Microscopy

HeLa cells were seeded into a 96-well, black-sided clear bottom plate at a density of 2.5 × 10^4^ in MEM media containing 20% (*v*/*v*) FBS and were given 24 h to adhere at 37 °C, 95% O_2_ and 5% CO_2_. The cells were then transfected with pEGFP-Sept2^WT^ or pEGFP-Sept2^P259A^ alongside pmCherry-α-tubulin using Dharmacon™ DharmaFECT™ kb transfection reagent. The cells were incubated for 24 h at 37 °C, 95% O_2_ and 5% CO_2_ with the plasmid-DharmaFECT™ complexes before the media was replaced with MEM containing 20% (*v*/*v*) FBS, supplemented with 160 nM nocodazole, for a further 16 h to allow for mitotic enrichment. Nocodazole was removed from the cells by carefully aspirating the media and gently washing the cells with PBS once. Once the PBS was removed, 150 μL of MEM lacking phenol red (GIBCO) supplemented with 10% (*v*/*v*) FBS was added in its place. Cells were examined immediately using a Nikon Eclipse Ti-E microscope equipped with a temperature-controlled humidified chamber (37 °C, 5% CO_2_), an Andor iXon EMCCD camera, spinning disc and motorised Piezo Z250 stage, with a 100× oil objective lens (NA 1.4) coupled with intermediate magnification (1.5×). Image acquisition was achieved using Andor Fusion software (software available to download at https://andor.oxinst.com/downloads/, accessed on 1 July 2022) (with a maximum EM gain of 100 and exposure time of 100 ms for each laser being used (either 561 nm or 488 nm for mCherry and EGFP-tagged constructs, respectively); each time sequence was created by imaging a cell every 60 s to 180 s for up to 150 min post-telophase from the point of metaphase onward. The end and start points of each Z-stack were selected manually depending on the cell, with 0.3 μm steps in all cases. Subsequent analysis was performed using Imaris X64 7.6.5.

## Data Availability

The mass spectrometry proteomics data have been deposited to the ProteomeXchange Consortium via the PRIDE [1] partner repository with the dataset identifier PXD042822.

## Supplementary Materials

The supporting information can be downloaded at https://www.mdpi.com/article/10.3390/ijms241311084/s1, references [70,71] are cited in Supplementary Materials.
