# Supplementary material for: Cyclophilin A Isomerisation of Septin 2 Mediates Abscission during Cytokinesis
Source: Int J Mol Sci. 2023 Jul 4;24(13):11084. doi: 10.3390/ijms241311084 (PMC10341793; doi:10.3390/ijms241311084)
Supplement: Supplementary file 1 [file ijms-24-11084-s001.zip › ijms-2425966-supplementary.pdf]

Supplementary Material

Cyclophilin A isomerisation of Septin 2 mediates abscission during cytokinesis

Rebecca L Gorry[1], Kieran Brennan[1], Paul TM Lavin[1], Tayler Mazurski[1], Charline Mary[3], David G Matallanas[2], Jean-François Guichou[3] and Margaret M Mc Gee[1,*]

Content:

Table S1 – Characterisation of proline residues within Sept2 as putative sites of CypA isomerisation.

Table S2 – Antibodies and plasmids

Table S3 – Primers used in site-directed mutagenesis (SDM)

Figure S1 – Over-expression of Sept2 in mammalian cells causes mitotic defects and chromosomal instability.

Video S1 – HeLa cells expressing pmCherry-α-tubulin complete abscission within 90 min post-telophase onset.

Video S2 – Expression of EGFP-Sept2^{P259A} significantly delays cytokinetic abscission in HeLa cells.

Video S3 – Expression of EGFP-Sept2^{WT}, together with endogenous Sept2, induces mitotic defects including multipolarity and arrest at the metaphase/anaphase transition in HeLa cells.

Sept2 Prolines	Sequence Motif	Location in Sept2	Exposed Proline	Evolutionary Conservation	Septin family conservation
SKQQ**P**TQFI	P-X-Pro-P	Pro6	-	Eutheria (96)	Sept2
QFIN**P**ETPG	H-X-Pro-P	Pro12	+/-	Amniota (92)	Sept2
NPET**P**GYVG	P-X-Pro-H	Pro14	+	Chordata (111)	Sept2
FANL**P**NQVH	P-X-Pro-P	Pro24	+	Fungi (63), Bilateria (22)	Sept2, Sept7
TDLY**P**ERVI	H-X-Pro-P	Pro64	-	Bilateria (86)	Sept2
ERVI**P**GAAE	H-X-Pro-H	Pro69	+/-	Actinopteri (57), Theria (27)	Sept2
VVDT**P**GYGD	C-X-Pro-H	Pro103 (G3 motif)	-	Opisthokonta (105), Candidatus Rokubacteria (2)	Sept1, Sept2, Sept3, Sept4, Sept5, Sept7, Sept9, Sept12
YFIS**P**FGH	H-X-Pro-H	Pro155	-	Amniota (62), Osteoglossocephalai (10)	Sept1, Sept2, Sept4, Sept5
HGLK**P**LDVA	H-X-Pro-H	Pro162	+	Bilateria (80), Saccharomyces ludwigii (1)	Sept2, Sept7
VNIV**P**VIAK	H-X-Pro-H	Pro179 (G4 motif)	-	Euteleostomi (64), Cyclophyllidea (2), Protostomia (3)	Sept2, Sept9
IYHL**P**DAE	P-X-Pro-C	Pro214	-	Euteleostomi (97), Stenosarchaea (2), Planctomycetaceae (3)	Sept2
KASI**P**FSVV	P-X-Pro-H	Pro236	-	Theria (84), Gymnophiona (2)	Sept1, Sept2, Sept4
GRLY**P**WGVV	H-X-Pro-A	Pro259 (SUD)	+	Deuterostomia (73), Arthropoda (24)	Sept2, Sept4, Sept5
EVEN**P**EHND	C-X-Pro-C	Pro268 (SUD)	-	Gnathostomata (72), Ecdysozoa (12)	Sept2

Table S1 – Characterisation of proline residues within Sept2 as putative sites of CypA isomerisation.

14 Sept2 proline residues are highlighted in bold, with the surrounding putative CypA binding motif underlined. Molecular modelling of Sept2 to determine exposed proline residues was performed using Molegro Molecular Viewer (at: http://molexus.io/molegro-molecular-viewer/) with the trimer Sept2-Sept6-Sept7 crystal structure determined by Sirajuddin, M (PDB ID: 2QAG) (Sirajuddin et al., 2007). Evolutionary conservation of each Sept2 proline residue was examined using the NCBI Basic Local Alignment Search Tool (BLAST®) protein-protein BLAST (BLASTp) function (Madden, 2013) (https://blast.ncbi.nlm.nih.gov/Blast.cgi). Sept2 family conservation was determined using Clustal Omega (https://www.ebi.ac.uk/Tools/msa/clustalo/) (Madeira et al., 2019). Four proline residues selected for experimental investigation as isomerase substrates (Pro24, Pro69, Pro162 and Pro259) are shaded in orange. ***H***: hydrophobic amino acid, ***X***: any amino acid, ***P***: polar amino acid, ***C***: charged amino acid, ***A***: amphipathic amino acid, ***G3/4***: guanine nucleotide-binding motifs, ***SUD***: septin-unique domain.

Table S2 – Antibodies and plasmids

Protein/Plasmid	Catalogue	Manufacturer	Application
AlexaFluor 488	A32731	ThermoFisher	IF
Alexa Fluor 594	A32742	ThermoFisher	IF
DyLight™ Fluor 688	10797775	Fisher Scientific	WB
DyLight™ Fluor 800	10733944	Fisher Scientific	WB
CypA	ab126738	Abcam	WB
GFP	ab290	Abcam	WB
GAPDH	MAB374	Millipore	WB
Sept2		Abcam	IF
α-tubulin	ab7291	Abcam	IF
pEGFP-C1	Discontinued	BD Biosciences, Clontech	WB, IF, IP
pEGFP-CypA^{WT}	-	Mc Gee Lab	WB, IF, IP
pEGFP-Sept2^{WT}	-	Mc Gee Lab	WB, IF
pEGFP-Sept2^{P24A}	-	Mc Gee Lab	WB, IF
pEGFP-Sept2^{P69A}	-	Mc Gee Lab	WB, IF
pEGFP-Sept2^{P162A}	-	Mc Gee Lab	WB, IF
pEGFP-Sept2^{P259A}	-	Mc Gee Lab	WB, IF
pD444-CypA^{WT}		ATUM	IMAC, SEC
pD444-CypA^{R55A}	-	Mc Gee Lab	IMAC, SEC

Table S3 – Primers used in site-directed mutagenesis (SDM)

Gene	Point Mutation	Primer	Primer Sequence (5'-3')
**EGFP-Sept2^{WT}**	P24A	Forward<br>Reverse	caaacctc**GCC**aatcaagttcaccgaaaatcagtg<br>aacttgatt**GGC**gaggtttgcaaatccaacatagc
	P69A	Forward<br>Reverse	agtcata**GCT**ggagcagcagaaaaaattgaaagaactg<br>gctgctcc**AGC**tatgactctttctgggtac
	P162A	Forward<br>Reverse	acctaag**GCC**ttagatgtggcgtttatgaagg<br>acatctaa**GGC**cttaagtccatgtccaaaagg
	P259A	Forward<br>Reverse	gcctctac**GCC**tggggtgttgtggaagtg<br>ca**GGC**gtagaggcggcctctgaccttctttc

Uppercase, bold: altered nucleotides

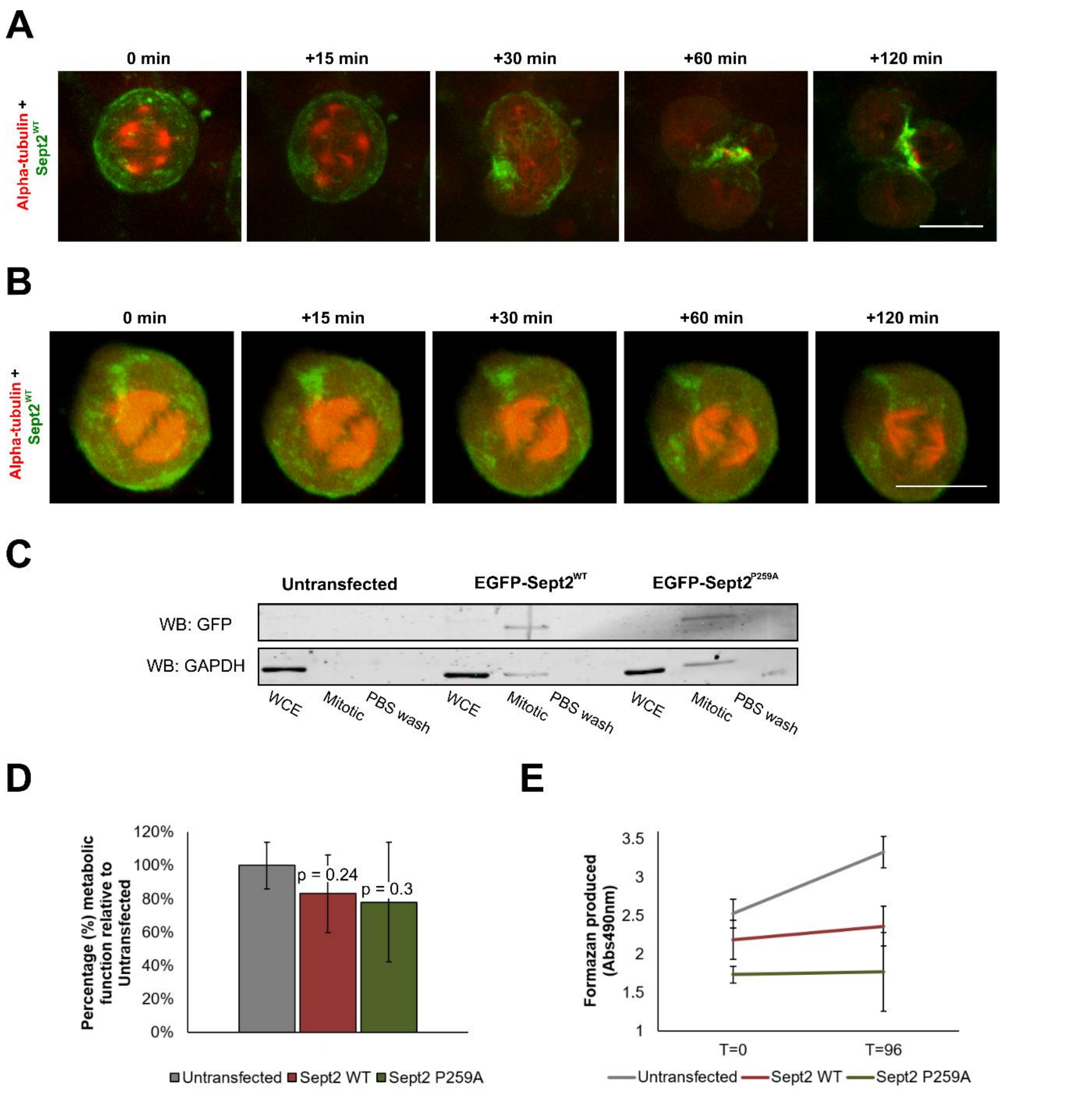

Figure S1 – Over-expression of Sept2 in mammalian cells causes mitotic defects and chromosomal instability.

A, **B**, HeLa cells were transfected with pmCherry-α-tubulin and pEGFP-Sept2^{WT} and enriched in mitosis by treatment with nocodazole (160 nM) for 16 hr. Cells were released from the nocodazole and imaged immediately using a Nikon Eclipse Ti-E microscope equipped with a temperature controlled, humidified chamber (37°C, 5% CO_2) and an 100X oil-immersion objective lens (1.4) coupled with intermediate magnification (1.5X). Z-stacks (0.3 µm slices, 20 µm in total) were acquired from metaphase/late anaphase onwards, every 60 s – 80 s for up to 150 min post-telophase. Scale bar: 15 µm. **C**, HeLa cells were left untransfected or transfected with pEGFP-Sept2^{WT} or pEGFP-Sept2^{P259A} and left for 24 hr. The media was collected ('*Mitotic*') and the cells were washed once ('PBS wash') before WCE were prepared and subjected to SDS-PAGE followed by Western Blot analysis. All lysates were probed with anti-GFP and anti-GAPDH primary antibodies. **D**, **E**, HeLa cells were either untransfected or transfected with pEGFP-Sept2^{WT} or pEGFP-Sept2^{P259A} and subjected to MTS analysis at T=0 hr (24 hr post-transfection) and T = 96 hr (120 hr post-transfection). **D**, The percentage of metabolic product generated

was graphed relative to the average values obtained for the untransfected HeLa cells, and p-values were derived from two-tailed, two-sample t-tests assuming unequal variance. **E**, Line graph highlighting the actual average absorbance readings obtained for each condition. Results in **D** and **E** are representative of biological triplicates.

Videos

Video S1 – HeLa cells expressing pmCherry-α-tubulin complete abscission within 90 min post-telophase onset.

HeLa cells were seeded into a 96-well, black-sided clear bottom plate and allowed to adhere for 24 hr prior to transfection with pmCherry-α-tubulin using DharmaFECT™ kb transfection reagent according to the manufacturer's instructions. Cells were incubated with the plasmid-DharmaFECT™ complexes for 24 hr before the transfection media was replaced for fresh, complete media supplemented with nocodazole (160 nM) for a further 16 hr. Cells were released into complete media lacking phenol red, and The cells were examined immediately using a Nikon Eclipse Ti-E microscope equipped with a temperature controlled humidified chamber (37°C, 5% CO_2); an Andor iXon EMCCD camera; spinning disk and motorised Piezo Z250 stage, with an 100X oil objective lens (NA 1.4) coupled with intermediate magnification (1.5X). Image acquisition was achieved using Andor Fusion software with a maximum EM gain of 100 and exposure time of 100 ms for the laser being used (561 nm). Each time sequence was created by imaging a cell from the point of metaphase onwards, every 60 s to 180 s for up to 150 min post-telophase. The end and start points of each Z-stack were selected manually depending on the cell, with 0.3 μm steps and 20 μm in total. Each frame represents the maximum projection determined for each Z-stack acquired, with a frame rate of 8 fps. Video is representative of 3 experimental replicates. Scale bar: 15 μm.

Video S2 – Expression of EGFP-Sept2^{P259A} significantly delays cytokinetic abscission in HeLa cells.

HeLa cells were seeded into a 96-well, black-sided clear bottom plate and allowed to adhere for 24 hr prior to transfection with pmCherry-α-tubulin (red) and pEGFP-Sept2^{P259A} (green) using DharmaFECT™ kb transfection reagent according to the manufacturer's instructions. Cells were incubated with the plasmid-DharmaFECT™ complexes for 24 hr before the transfection media was replaced for fresh, complete media supplemented with nocodazole (160 nM) for a further 16 hr. Cells were released into complete media lacking phenol red, and The cells were examined immediately using a Nikon Eclipse Ti-E microscope equipped with a temperature controlled humidified chamber (37°C, 5% CO_2); an Andor iXon EMCCD camera; spinning disk and motorised Piezo Z250 stage, with an 100X oil objective lens (NA 1.4) coupled with intermediate magnification (1.5X). Image acquisition was achieved using Andor Fusion software with a maximum EM gain of 100 and exposure time of 100 ms for the laser being used (561 nm and 488 nm). Each time sequence was created by imaging a cell from the point of metaphase onwards, every 60 s to 180 s for up to 150 min post-telophase. The end and start points of each Z-stack were selected manually depending on the cell, with 0.3 μm steps and 20 μm in total. Each frame represents the maximum projection determined for each Z-stack acquired, with a frame rate of 8 fps. Video is representative of 3 experimental replicates. Scale bar: 15 μm.

Video S3 – Expression of EGFP-Sept2^{WT}, together with endogenous Sept2, induces mitotic defects including multipolarity and arrest at the metaphase/anaphase transition in HeLa cells.

HeLa cells were seeded into a 96-well, black-sided clear bottom plate and allowed to adhere for 24 hr prior to transfection with pmCherry-α-tubulin (red) and pEGFP-Sept2^{WT} (green) using DharmaFECT™ kb transfection reagent according to the manufacturer's instructions. Cells were incubated with the plasmid-DharmaFECT™ complexes for 24 hr before the transfection media was replaced for fresh,

complete media supplemented with nocodazole (160 nM) for a further 16 hr. Cells were released into complete media lacking phenol red, and The cells were examined immediately using a Nikon Eclipse Ti-E microscope equipped with a temperature controlled humidified chamber (37°C, 5% CO_2); an Andor iXon EMCCD camera; spinning disk and motorised Piezo Z250 stage, with an 100X oil objective lens (NA 1.4) coupled with intermediate magnification (1.5X). Image acquisition was achieved using Andor Fusion software with a maximum EM gain of 100 and exposure time of 100 ms for the laser being used (561 nm and 488 nm). Each time sequence was created by imaging a cell from the point of metaphase onwards, every 60 s to 180 s for up to 150 min post-telophase. The end and start points of each Z-stack were selected manually depending on the cell, with 0.3 μm steps and 20 μm in total. Each frame represents the maximum projection determined for each Z-stack acquired, with a frame rate of 8 fps. Video is representative of 3 experimental replicates. Scale bar: 15 μm.

References

Madden, T. 2013. The BLAST sequence analysis tool. *BLAST Seq. Anal. Tool*.

Madeira, F., Y.M. Park, J. Lee, N. Buso, T. Gur, N. Madhusoodanan, P. Basutkar, A.R.N. Tivey, S.C. Potter, R.D. Finn, and R. Lopez. 2019. The EMBL-EBI search and sequence analysis tools APIs in 2019. *Nucleic Acids Res.* 47:W636–W641. doi:10.1093/nar/gkz268.

Sirajuddin, M., M. Farkasovsky, F. Hauer, D. Kühlmann, I.G. Macara, M. Weyand, H. Stark, and A. Wittinghofer. 2007. Structural insight into filament formation by mammalian septins. *Nature*. 449:311–315. doi:10.1038/nature06052.
